# B7H1 Expression and Epithelial-To-Mesenchymal Transition Phenotypes on Colorectal Cancer Stem-Like Cells

**DOI:** 10.1371/journal.pone.0135528

**Published:** 2015-08-18

**Authors:** Yidan Zhi, Zhirong Mou, Jun Chen, Yujun He, Hui Dong, Xiaolan Fu, Yuzhang Wu

**Affiliations:** 1 Institute of Immunology of PLA, Third Military Medical University, Chongqing, China; 2 Department of General surgery, Southwest Hospital, Chongqing, China; 3 Department of General surgery, Daping Hospital, Chongqing, China; Istituto Nazionale Tumori, ITALY

## Abstract

Cancer stem cells (CSCs) can invade and metastasize by epithelial-to-mesenchymal transition (EMT). However, how they escape immune surveillance is unclear. B7H1 is crucial negative co-stimulatory molecule but little information about whether it works in CSCs. Therefore, we determined the expression of B7H1 and EMT-associated markers in colorectal cancer stem-like cells to investigate a possible immunoevasion way of CSCs. We enriched CD133^+^ colorectal cancer cells which manifested the CSCs-like properties such as higher levels of other stem cell markers Oct-4 and Sox-2, tumor sphere forming ability and more tumorigenic in NOD/SCID mice. These CD133^+^ cells possess EMT gene expression profile including higher level of Snail, Twist, vimentin, fibronectin and lower level of E-cadherin. Moreover, CD133^+^ cells in both cell line and colorectal cancer tissues expressed high level of negative co-stimulate molecule B7H1. Furthermore, some B7H1^+^ cancer cells also showed the characteristic of EMT, indicating EMT cells could escape immune attack during metastasis. B7H1 expression and EMT phenotypes on CSCs indicates a possible immunoevasion way.

## Introduction

Colorectal cancer is the third most commonly diagnosed cancer in males and the second one in females [1], but advancements of anti-cancer therapy have been made limitedly in the past 50 years. Failure of anti-cancer therapy is attributed to a subpopulation of cancer cells called cancer stem cells (CSCs), which are the putative cancer-initiating cells with the characteristics of normal stem cells, such as self-renewal, multipotency and limitless proliferation potential [2]. Moreover, CSCs are thought to be crucial for drug-resistance [3]. Therefore, it is believed that CSCs are the “seeds” of cancer formation and difficult to be eliminated. Colorectal CSCs have also been isolated and characterized based on CSCs markers such as CD133 [4–9].

CSCs play a crucial role in cancer invasion and metastasis. To understand how cancer cells metastasize, the role of the epithelial-to-mesenchymal transition (EMT) has been extensively studied over the past decade. EMT confers invasive and metastatic characteristics, resistance to therapies, and CSCs phenotypes on cancer cells in experimental models [10–15]. Cancer cells undergoing EMT downregulate the proteins associated with cell adhesion, such as E-cadherin, and upregulate proteins expressed on mesenchymal cells, such as vimentin, N-cadherin and fibronectin [13], and transcription factors including *Snail*, *Twist*, and *Slug* as well [16]. EMT also facilitates cancer cell survival after treatment with anti-cancer drugs, which target receptors on epithelial cells [12, 17]. In addition, induction of EMT in cancer cells with drugs or overexpression of EMT transcription factors results in acquisition of mesenchymal properties and in expression of stem-cell markers [18–20]. On the other hand, cancer cells following treatment with anti-cancer drugs, which have been shown to enrich CSCs, manifest the phenotypes and gene expression like EMT [21]. These findings indicate the close association between CSCs and the acquisition of EMT. However, a majority of pathologists are still refractory to the EMT theory because definitive proof of EMT happening in human tumors is lacking so far.

CSCs possess intrinsic biological characteristics to form tumor and may invade tissues through EMT. But it is unclear that how they evade immune surveillance for final survival in immunocompetent hosts. Immunoevasion may help CSCs to survive and then form tumor [3]. Previous reports have suggested inherent connections between immune suppression and EMT, such as that Snail-induced EMT induced regulatory T cells and impaired dendritic cells [22]. Taken together, we hypothesize immunoevasion is important for CSCs that undergo EMT through paraneoplastic inflammation region without immune clearance and then implement invasion and metastasis. However, data is still scarce of the immunoevasion mechanisms in CSCs [3].

B7H1, a ligand of programmed cell death 1 (PD-1), has been well-known as a crucial co-stimulatory molecule and plays an important role in the induction and maintenance of peripheral tolerance [23]. B7H1 is upregulated on considerable kinds of cancer cells which offers negative signals and leads to immunosuppression through PD-1-B7H1 interaction between cancer cells and T cells [24, 25], resulting in tumor-infiltrating T cells dysfunction and Treg recruitment [26]. These traits make B7H1 become a promising target to control cancer. Nevertheless, B7H1 expression on CSCs is not known well in colorectal cancer. Thus, we detected B7H1 expression in colorectal cancer in this study and showed B7H1 expression and EMT phenotypes on colorectal cancer stem-like cells, which might be mechanisms for CSCs to escape immune surveillance and invade distant tissues.

## Materials and Methods

### Ethics statement

The human colorectal cancer tissues were obtained from the Southwest Hospital under ethical protocols approved by the Ethics Committee of the Third Military Medical University, Chongqing, China. All patients provided written informed consent. Animal experiments were approved by the Institutional Animal Care and Use Committee of the Third Military Medical University. All procedures were in compliance with the National Institute of Health Guide for Care and Use of Laboratory Animals.

### Cancer tissues and cell Line

The human colorectal tumors were histopathologically evaluated and classified by the Tumor-Node-Metastasis (TNM) staging system (Table 1). The colon cell line HT29 cells (Catalog number: TCHu 103, purchased on May 29th, 2010 from the Cell Bank of the Chinese Academy of Sciences, Shanghai, China) were cultured in McCoy’s 5A (Invitrogen, Carlsbad, CA, USA) supplemented with 10% fetal bovine serum (FBS) (Gibco, Grand Island, NY, USA).

**Table 1:** Patients’ information and cancer characteristics.

*Case*	*Age/Sex*	*Site*	*TMN stage*
1	69/M	Right	T2N0M0
2	58/M	Right	T3N0M0
3	74/M	Left	T3N0M0
4	59/F	Right	T3N0M0
5	63/M	Right	T3N0M0
6	59/F	Rectum	T3N0M0
7	37/F	Rectum	T3N0M0
8	68/M	Sigma	T3N0M0
9	56/F	Left	T3N0M0
10	74/F	Sigma	T3N0M0
11	69/M	Left	T3N0M0
12	56/F	Right	T3N0M0
13	56/M	Right	T3N0M0
14	59/F	Right	T3N0M0
15	58/F	Left	T4N0M0
16	76/M	Sigma	T4N2M1
17	76/M	Sigma	T4N2M0
18	55/M	Right	T4N2M0
19	70/M	Sigma	T4N2M0
20	49/M	Right	T4N2M1

### Magnetic cell sorting

HT29 cells were harvested at exponential growth phase with 0.25% trypsin (contained EDTA) then sorted by CD133 Cell Isolation Kit (Miltenyi Biotec GmbH, Bergisch Gladbach, Germany) according to the instructions of manufacturer. The dead cells were removed by Dead Cell Removal Kit (Miltenyi Biotec) before sorting. The CD133^+^ and CD133^-^ fractions were collected separately and stained with PE-conjugated human CD133/2 (293C3) antibody (Miltenyi Biotec). PE-conjugated mouse IgG2b (Miltenyi Biotec) was used as isotype control antibody. The cell purity was assessed by flow cytometry (BD FACSCanto Ⅱ, San Jose, CA, USA).

### Tumorigenic assays in mice

NOD/LtSz-scid/scid (NOD/SCID) mice (Purchased from Beijing Laboratory Animal Research Center, China) were bred and maintained in Individual Ventilated Caging System under conditions approved by the Institutional Animal Care and Use Committee of the Third Military Medical University. CD133^+^ and CD133^-^ cells were sorted and counted before inoculation. Cancer cells were suspended in a 1:1 mixture of media and matrigel (BD Biosciences, Bedford, UK) and injected subcutaneously into the flanks of 4 mice (about 8–10 weeks of age). Each flank was injected 1×10^4^ cells. The growth of tumors was monitored weekly and measured the volume by length×width×width/2. All mice were sacrificed after 7 weeks transplantation. The xenografts were removed, fixed with 10% buffered formalin, and stained with hematoxylin and eosin(HE).

### Sphere formation assay

The sorted CD133^+^ and CD133^-^ cells were cultured in serum-free medium (NS-A basal medium, Stemcell Technologies, Vancouver, BC, Canada) plus 10% NS-A Proliferation Supplements (Human) (Stemcell Technologies), epidermal growth factor(EGF)(20 ng/mL) (Stemcell Technologies), basic fibroblast growth factor (bFGF)(10 ng/mL) (Stemcell Technologies) and heparin (2 μg/mL) (Invitrogen). The culture medium was replaced every 3 days. The images of spheres were captured after 20 days by microscope and counted numbers under 100×magnification in random visual fields. Some spheres were stained with PE-conjugated CD133/2 (293C3) antibody (Milteny Biotect). Some tumor spheres were dissociated by Collagenase Type Ⅳ(Sigma-Aldrich, St Louis, MO, USA) and then cultured in serum-free medium as mention above to determine whether spheres could form again.

### RNA extraction, cDNA synthesis, and Q-PCR

CD133^+^ and CD133^-^ HT29 cells were collected respectively and added Tripure (Roche Diagnostics, Indianapolis, IN, USA) to extract RNA. 500 ng RNA for each sample was reversely transcribed in a 20 μL reaction mixture (TOYOBO Bio-Technology, Shanghai, China). Expression level of each gene was determined by real-time quantitative PCR (Q-PCR). Level of product was determined by SYBR Green (TAKARA Biotechnology, Dalian, China). Q-PCR was performed by Mx3000P qPCR system (Agilent Technologies) with the following program: initial denaturation for 2 minutes at 95°C followed 45 cycles of PCR (95°C for 10 seconds, 60°C for 30 seconds, 72°C for 30 seconds). After that one cycle designed by Mx3000P software (95°C for 60 seconds, 55°C for 30 seconds, 95°C for 30 seconds) was added for melting curve analysis. Primer sequences were listed in Table 2. Glyceraldehyde phosphate dehydrogenase(GAPDH) was used as the housekeeping gene control.

**Table 2:** Primers used for Real-time PCR.

*Gene name*	*Forward(5’-3’)*	*Reverse(5’-3’)*
Oct-4	CCTGAAGCAGAAGAGGATC	CGTTTGGCTGAATACCTT
Sox-2	CCAGCTCGCAGACCTACAT	ACTTGACCACCGAACCCA
Snail	CCAGTGCCTCGACCACTATG	GCAGCTCGCTGTAGTTAGGCTTC
Twist	GGCCAGGTACATCGACTTCC	CCGCTCGTGAGCCACATA
E-cadherin	CAGCACGTACACAGCCCTAA	ACCTGAGGCTTTGGATTCCT
Vimentin	GACAATGCGTCTCTGGCACGTCTT	TCCTCCGCCTCCTGCAGGTTCTT
Fibronectin	CAGGATCACTTACGGAGAAACAG	GCCAGTGACAGCATACACAGTG
GAPDH	GCACCGTCAAGGCTGAGAAC	TGGTGAAGACGCCAGTGGA

### Immunofluorescence staining

The frozen sections of cancer tissues were stained according to online protocol (http://www.ihcworld.com/_protocols/general_IHC/immunofl.htm). Antibodies against human CD133, B7H1, E-cadherin and vimentin were used to determine the expression of EMT markers on CD133 positive or B7H1 positive cells. Similarly, antibodies against human CD133, B7H1 and EpCAM were used to detect CD133 and B7H1 expression on cancer tissues. The details of antibodies selection were listed in Table 3. Secondary antibodies were purchased from Beyotime Institute Biotechnology, Shanghai, China. Diamidino-phenyl-indole (DAPI) (Beyotime Biotechnology) was used to stain nucleus. Controls included: 1) Blank: used antibody dilution buffer (5% bovine serum albumin in phosphate-buffer saline, pH 7.2) only; 2) Negative control: used antibody dilution buffer plus secondary antibodies; 3) Isotype antibody control: used IgG from antibody species diluted by antibody dilution buffer plus secondary antibodies. Controls were used to avoid nonspecific fluorescence.

**Table 3:** Antibodies list of immunofluorescence staining.

Fig. No.	Primary antibodies	Secondary antibodies
Fig 3A	Mouse anti-human CD133 (Miltenyi Biotec)	Goat anti-mouse IgG-Cy5
	Rabbit anti-human E-cadherin (Santa Cruz)	Goat anti-rabbit IgG-FITC
Fig 3B	Rabbit anti-human CD133 (Abcam)	Goat anti-rabbit IgG-FITC
	Mouse anti-human vimentin (Santa Cruz)	Goat anti-mouse IgG-Cy5
Fig 3C	PE-conjugated mouse anti-human CD133 (Miltenyi Biotec)	-
	Rabbit anti-human E-cadherin (Santa Cruz)	Goat anti-rabbit IgG-FITC
	Mouse anti-human vimentin (Santa Cruz)	Goat anti-mouse IgG-Cy5
Fig 4A	PE-conjugated mouse anti-human CD133 (Miltenyi Biotec)	-
	Mouse anti-human B7H1 (provided by Prof. Lieping Chen, Yale University, USA)	Goat anti-mouse IgG-FITC
Fig 4B (C)S1 Fig	Rabbit anti-human CD133 (Abcam)	Goat anti-rabbit IgG-Cy3
	Mouse anti-human B7H1 (as in Fig 4A)	Goat anti-mouse IgG-FITC
	APC-conjugated mouse anti-human EpCAM (Miltenyi Biotec)	-
Fig 5A	Mouse anti-human B7H1 (as in Fig 4A)	Goat anti-mouse IgG-Cy5
	Rabbit anti-human E-cadherin (Santa Cruz)	Goat anti-rabbit IgG-FITC
Fig 5B	Rabbit anti-human B7H1 (Santa Cruz)	Goat anti-rabbit IgG-FITC
	Mouse anti-human vimentin (Santa Cruz)	Goat anti-mouse IgG-Cy5

HT29 cells staining protocol was similar to cancer tissues, but before that cells were seeded onto poly-L-lysine (Sigma-Aldrich) precoated-cover slips, cultured in McCoy’ 5A medium for 24 h and then fixed with Fixation and Permeabilization Solution (BD Biosciences).

After staining, all slides were kept in humidified chambers away from light at 4°C till image capture.

### Images capture and expression analysis

All confocal images were captured by laser scanning confocal fluorescence microscope (Leica TCS-SP5, Germany). Controls were used to adjust proper capture condition to avoid nonspecific fluorescence interference. The same marker in different slides was detected under the same condition. Expression rates were measured by counting corresponding positive cells in the capture images. Each slide was measured more than 5 fields and counted at least 1000 cells.

### Statistical analysis

The differences of xenograft volumes, sphere numbers and mRNA expression level between CD133^+^ and CD133^-^ cells were analyzed by Student’s *t*-test. A *p*-value<0.05 was considered significant difference. All statistical analyses were performed using SPSS software (version 13.0).

## Results

### CD133^+^ cells possess more tumorigenic potential and tumor sphere forming ability

**Fig 1:**
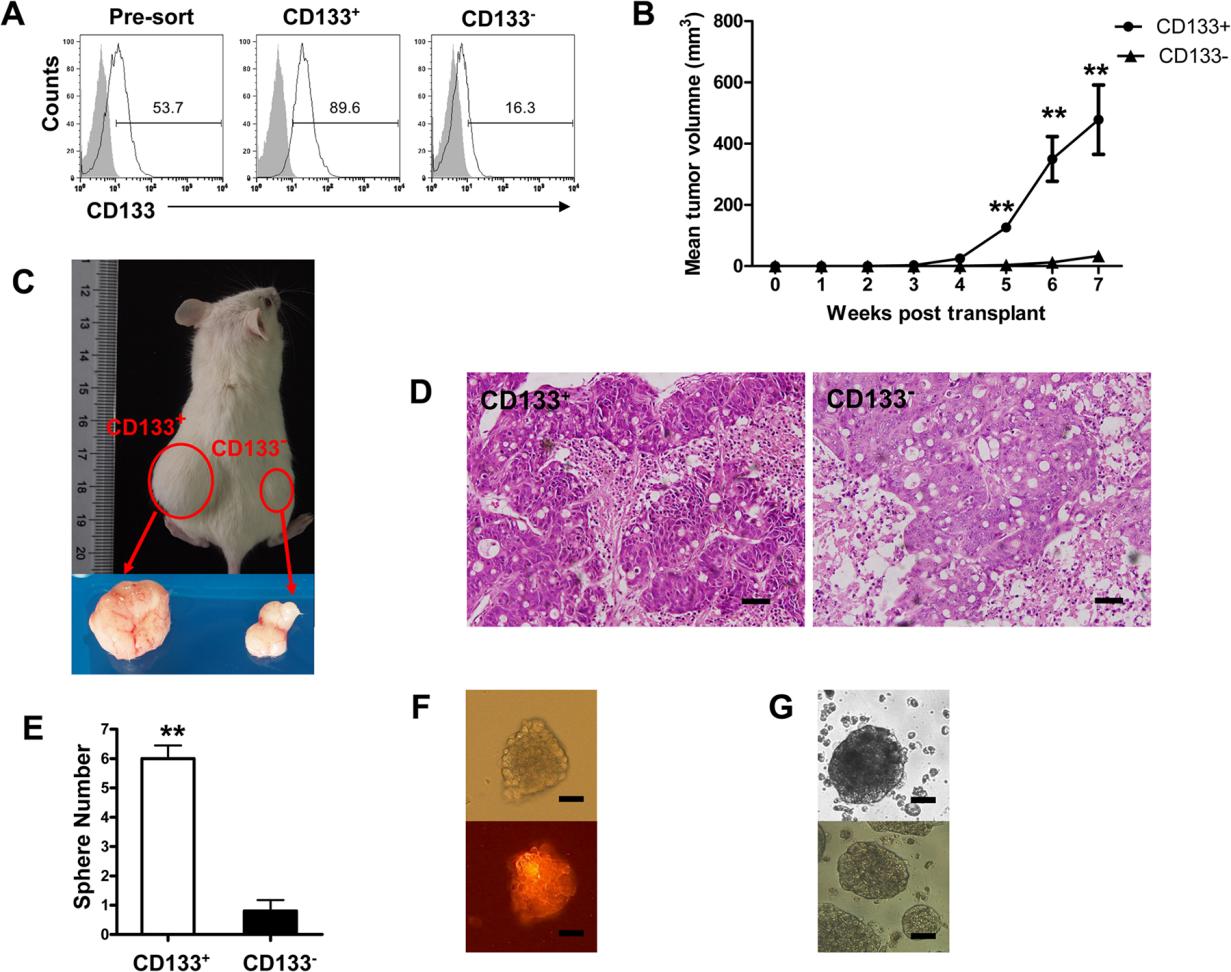
CD133^+^ HT29 cells have higher tumorigenic potential and tumor sphere forming ability. HT29 cells were purified with CD133 magnetic beads. The purity of CD133^+^ cells was determined by flow cytometry (A). 1×10^4^ CD133^+^ and CD133^-^ cells were subcutaneously injected into the different flanks of NOD/SCID mice. Growth curve of xenograft tumors in NOD/SCID mice shows different capability of tumorigenesis in two subpopulations (B). The kinetics of tumor size was monitored once a week (n = 4, mean ± SD, **, *p<*0.01).A representative of mice is shown at 7 weeks after inoculation (C). The sections of xenografts (D) from CD133^+^ cells (left, 200× magnification) and CD133^-^ cells (right, 200× magnification) were analyzed with hematoxylin and eosin stain (bar = 100 μm). CD133^+^ cells show much more powerful of sphere formation than CD133^-^ ones (E). The histogram shows numbers of spheres derived from different subpopulations (**, *p<*0.01). Tumor spheres express CD133 (F, top: neutral light; bottom: fluorescence, red; bar = 100 μm). After dissociation, cells formed spheres again (G, bottom) in serum-free culture medium like their elder generation (G, top) (bar = 100 μm).

As the debates of CSCs markers still exist [27, 28], we checked the cancer stem-like characteristics of sorted CD133^+^ cells first. CD133^+^ and CD133^-^ HT29 cells were collected for determining the cell tumorigenicity. The percentage of CD133^+^ cells was about 90% (Fig 1A). Ten thousands of CD133^+^ and CD133^-^ were subcutaneously injected into the different flanks of NOD/SCID mice. From 5 weeks after injection, the tumors derived from CD133^+^ cells started to be significantly larger than the ones derived from CD133^-^ cells (Fig 1B and 1C). The CD133^+^ cell xenografts showed malignant histology like colon cancer (Fig 1D), suggesting that CD133^+^ cells are more tumorigenic. In addition, sphere formation is one of traits of CSCs [4], we determined sphere formation of CD133^+^ cells and found that CD133^+^ cells formed significantly more tumor spheres than CD133^-^ cells in serum-free culture medium *in vitro* (Fig 1E). Moreover, almost all cells in CD133^+^ cells derived tumor spheres expressed high level of CD133 (Fig 1F). In addition, after cell dissociation with collagenase, the cells could form tumor spheres again in serum-free medium (Fig 1G), indicating the self-renewal capacity of CD133^+^ cells. Overall, these results indicated that CD133^+^ cells possessed CSCs-like properties.

### CD133^+^ HT29 cells express both stem cell-associated and EMT-associated molecules

**Fig 2:**
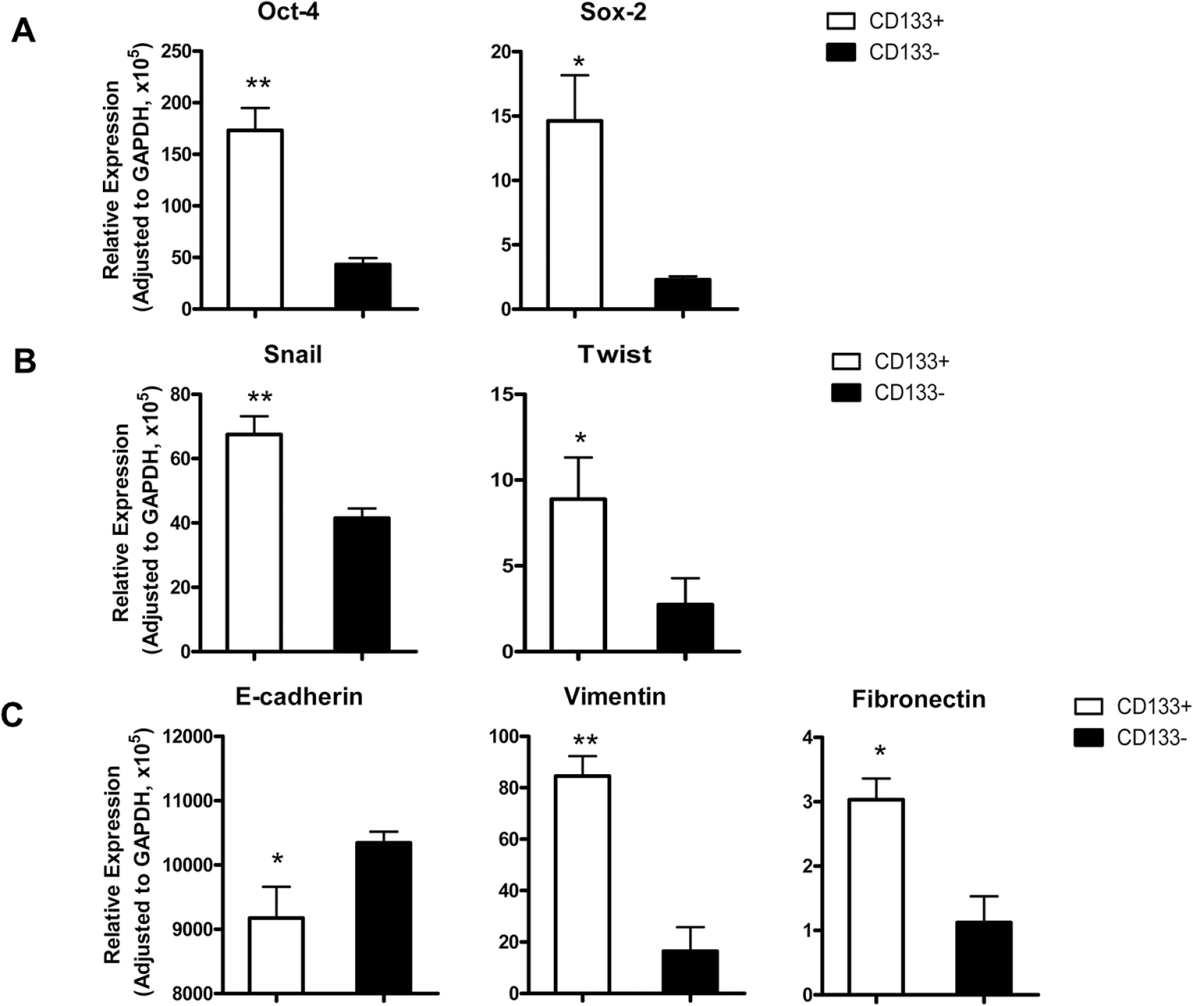
mRNA levels of stem cells markers and EMT associated molecules in CD133^+^ and CD133^-^ HT29 cells. Relative mRNA expression levels of stem cell markers Oct-4 and Sox-2 (A), EMT associated transcription factors Twist and Snail (B), and EMT markers E-cadherin, fibronectin, and vimentin (C) were determined by Real-time quantitative PCR and normalized to GAPDH expression. *, *p<*0.05; **, *p<*0.01.

CSCs are the putative cancer-initiating cells with the characteristics of normal stem cells expressing pluripotency markers, such as Sox-2 and Oct-4 [29–31]. To determine whether CD133^+^ HT29 cells also express those stem cell-associated factors, the transcriptional level of Oct-4 and Sox-2 expression was measured. As expected, two stem cell markers Oct-4 and Sox-2 were expressed significantly higher in CD133^+^ subpopulation (*p*<0.05) than CD133^-^ cells (Fig 2A), which is consistent with Oct-4 and Sox-2 overexpression in poorly differentiated tumors [32].

Previous study showed that EMT is involved in the conversion of early stage tumors into invasive malignancies [33]. Interestingly, accumulating evidences indicate that induction of EMT phenotypes by different factors also induces CSC-like cells [15]. However, it is not clear whether CSCs display EMT phenotypes. To address this question, expression of EMT-associated molecules in HT29 cells was detected, including transcription factors Snail and Twist, epithelial protein E-cadherin, mesenchymal markers vimentin and fibronectin. CD133^+^ cells expressed significantly higher levels of Snail, Twist, vimentin and fibronectin than CD133^-^ cells (*p<*0.05) (Fig 2B and 2C). Conversely, E-cadherin expression in CD133^+^ cells was significantly lower (*p* <0.05) (Fig 2C), indicating CD133^+^ cells manifested EMT-associated characteristics.

### CD133^+^ cells in patient tissues display EMT phenotypes

**Fig 3:**
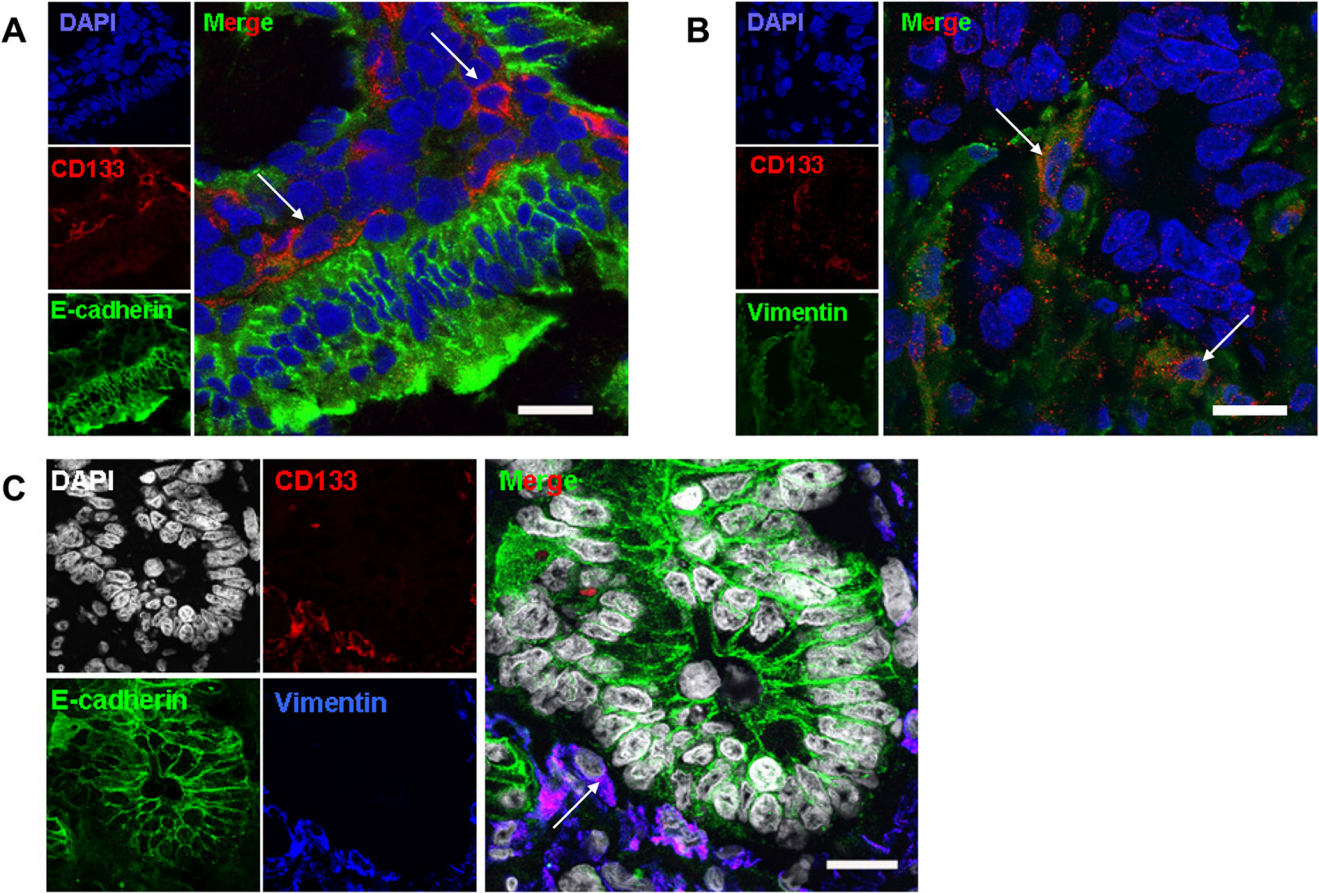
CD133^+^ cancer cells have the EMT phenotypes in colorectal cancer tissues. The expression of CD133 (red), E-cadherin (green) (A) or CD133 (red) and vimentin (green) (B) or CD133 (red), E-cadherin (green) and vimentin (blue) (C) were determined by immunofluorescence staining. The nucleuses were stained with DAPI (A, B: blue; C: grey). It is shown a representative of colon moderately differentiated adenocarcinoma, T3N0M0. (Bar = 20 μm)

Although EMT was widely studied *in vitro*, the key question about EMT was that the direct evidence of this phenomenon happening in human cancer tissues was lacking. To address this issue, expression of CD133 and EMT-phenotypic molecules was analyzed in human colorectal cancer tissues. CD133 was expressed dispersedly beside the adenocarcinoma nest (Fig 3) but not in paraneoplastic tissue (S1 Fig). Interestingly, we found that some CD133^+^ cells expressed vimentin (Fig 3B) but not E-cadherin in 13/20 detected human colorectal cancer tissues (Fig 3A). By staining these three molecules on the same section, we confirmed a portion of CD133^+^ cells in different samples (20%-40% in total CD133^+^ cells) showed this phenotype (Fig 3C). It indicated the direct evidence that some CD133^+^ cancer stem-like cells had EMT phenotypes in human cancer tissues.

### Co-expression of B7H1 and CD133 in HT29 cell line and colorectal cancer tissues

**Fig 4:**
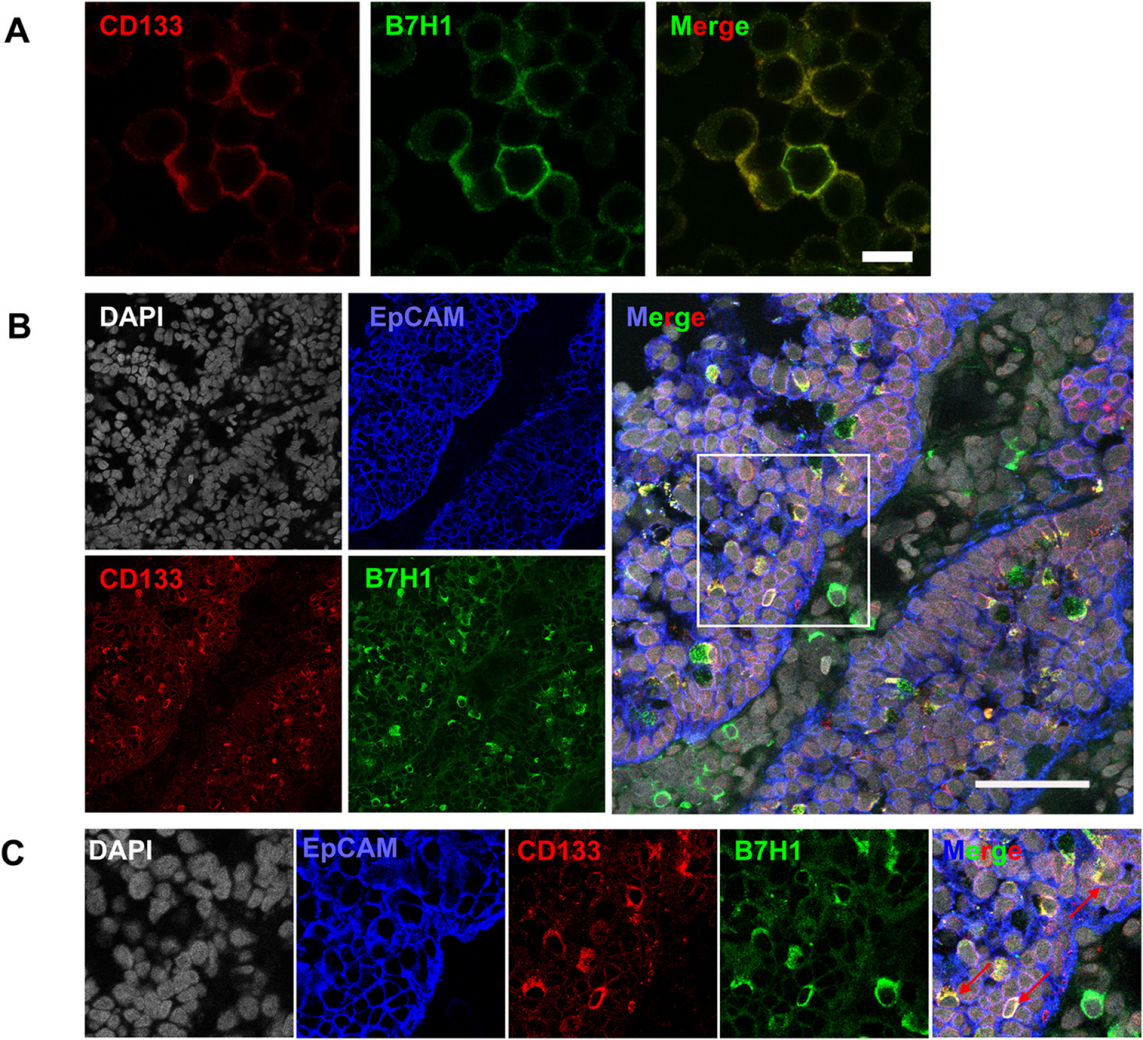
Co-expression of B7H1 and CD133 in HT29 cells and colorectal cancer tissues. The expression of CD133 (red) and B7H1 (green) in HT29 cells (A) (Bar = 20 μm), and expression of EpCAM (blue), CD133 (red) and B7H1 (green) in colorectal cancer tissues (B) (Bar = 50 μm) were determined by immunofluorescence staining. The nucleuses were stained with DAPI (grey). The rectangle area in the image is enlarged (C) and shows a typical co-expression of CD133 and B7H1 (indicated with arrows). It is shown a representative rectal moderately differentiated adenocarcinoma, T3N0M0.

To investigate the mechanism how CSCs escape immune surveillance, B7H1 expression was determined in HT29 cell line and colorectal cancer tissues. We found that co-expression of B7H1 and CD133 was ubiquitous in HT29 cells (Fig 4A). To further determine B7H1 expression in patient tissues, colorectal cancer tissues were collected. EpCAM, a luminal epithelial marker, was used to distinguish cancer nests and stromal region [34]. We found that B7H1 was expressed mainly in cancer cells in more than half (11/20) of colorectal cancer tissues (Table 4). More interestingly, about 50% of CD133 expressing cancer cells expressed B7H1 (Fig 4B and 4C, Table 4). Moreover, B7H1 and CD133 were always co-expressed in EpCAM^+^ cells (Fig 4B and 4C). It indicates CD133^+^ CSCs might escape immune surveillance by expressing inhibitory molecule B7H1.

**Table 4:** B7H1 and CD133 expression on human colorectal cancer tissues.

*TMN stage*	*Number of samples tested*	*Cases of B7H1 expressed on cancer cells*	*Percentage of B7H1 expression on CD133* ^*+*^ *cells*
T2M0N0	1	0	-
T3M0N0	13	7	(53±23)^##^
T4M0-2N0-2	6	4	(45±29)^$$^

##: data was obtained from 7 cases of B7H1 expressed on cancer cells in T3M0N0 stage.

$$: data was obtained from 4 cases of B7H1 expressed on cancer cells in T4M0-2N0-2 stage.

### B7H1 expressing cells display EMT phenotypes in colorectal tissues

**Fig 5:**
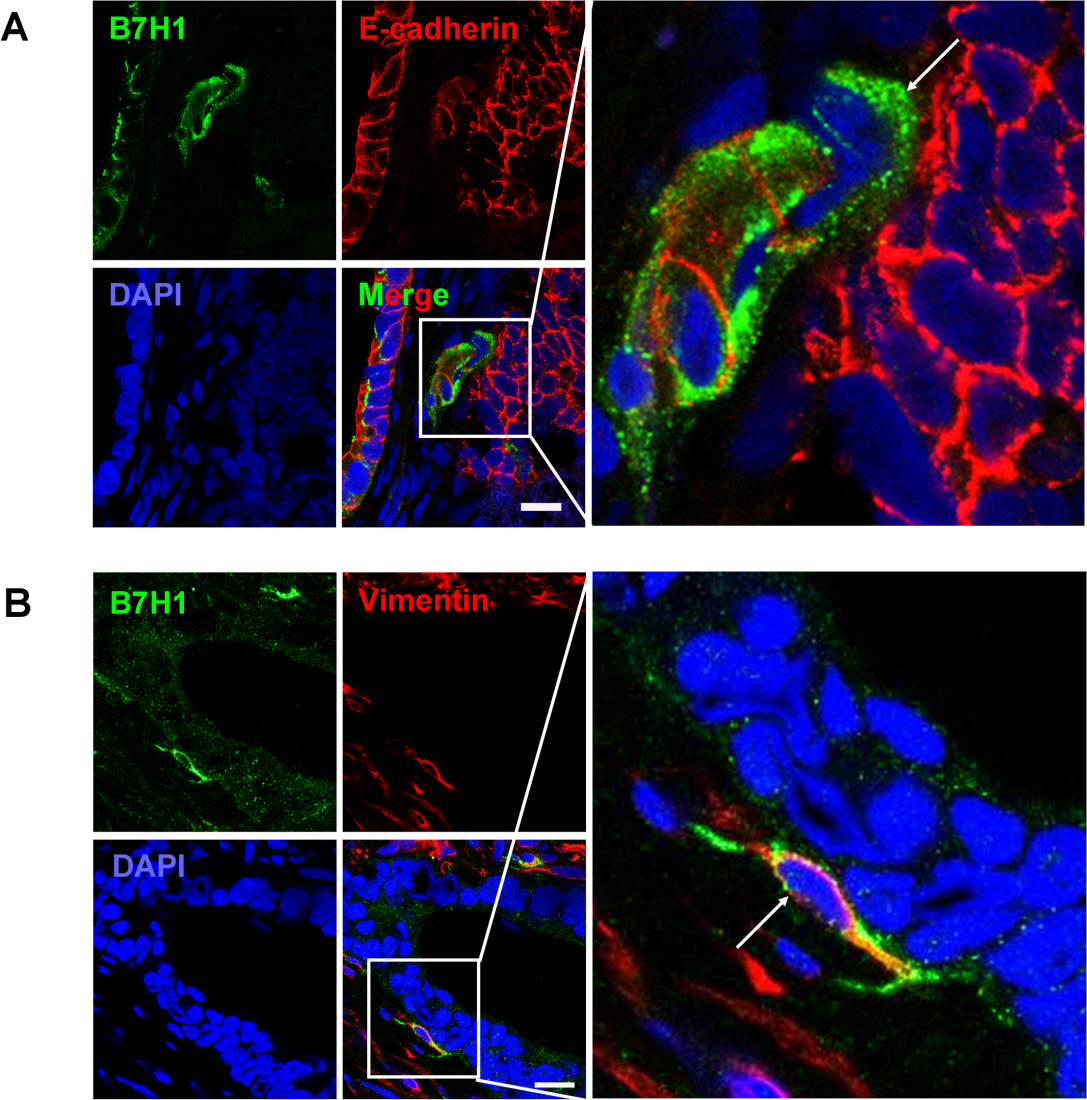
Some B7H1 expressing cells manifest the feature like EMT in colorectal cancer tissues. The expression of B7H1 (green) and E-cadherin (red) (A) or B7H1 (green) and vimentin (red) (B) were determined by immunofluorescence staining. One of B7H1 expressing cells is indicated with an arrow. The nucleuses were stained with DAPI (blue). It is shown a representative of colon moderately differentiated adenocarcinoma, T3N0M0. (Bar = 20 μm)

We found that B7H1 was expressed on CD133^+^ cells and might play a role in immunoevasion of cancer stem-like cells. It would be interesting to know whether B7H1 expression is related to EMT. To address this directly, B7H1 expression in EMT phenotypic cells was determined in 11 human colorectal cancer samples in which CD133 was co-expressed with B7H1. Similar to previous studies [35], B7H1 was expressed on cancer cells especially on the edge of cancer (Fig 5). In these cases, especially where it looked like tumor budding, we found that 20%-40% of B7H1expressing cancer cells down-regulated E-cadherin (Fig 5A). Furthermore, 10%-25% of B7H1^+^ cancer cells expressed vimentin, a mesenchymal marker (Fig 5B), indicating EMT phenotype exist in certain B7H1^+^ cells. Taken together, it suggested that overexpression of B7H1 might be one of immunoevasive mechanisms not only for CD133^+^ CSCs, but also for transient EMT phenotypic cancer cells.

## Discussion

CSCs are considered to be the “seeds” of cancers and can be enriched by cell sorting based on some specific surface markers. Though the markers of CSCs are still disputable, cancer cells expressing certain specific molecules display CSCs-like the properties [9]. CD133 is one of markers of hematopoietic stem cells and considered to be a CSCs marker in colorectal cancer [5–7, 27, 28, 36]. In this study, CD133^+^ cells had CSCs-like characteristics by showing capability of sphere forming and tumorigenicity, expressing higher levels of stem cell markers Oct-4 and Sox-2 which were overexpressed in poorly differentiated tumors [29, 31]. Moreover, these cells manifested EMT gene expression profile, which had been considered to be an important peculiarity linking to CSCs [15].

Our results of the CSCs-like traits of CD133^+^ cells were consistent with previous studies [5–7]. In contrary, there are some other results showed that CD133 negative cells possessed CSCs-like properties instead [27, 28]. For example, a CD133 negative cell line (NANK) was established from xenografts which derived from fresh surgical samples of human colonic primary and its ovarian metastatic cancer tissues transplanted into NOD/SCID mice [28]. NANK cells possessed CSCs-like characteristics such as sphere formation and tumorigenicity in NOD/SCID mice. Compared with our study, it can be found that the origin of cancer cells were quite different, which might interfere the consistency of results. Moreover, NANK cells differed from their original cancer as well, such as that CD133 and CD44 were expressed in original cancer but rather weakly in NANK. Therefore, it seems that this contrast implies the complex of cancer rather than the mistakes of research methods. Though it’s not rigorous enough to use CD133 alone to identify CSCs, it’s confirmed that CSCs were enriched in CD133^+^ subpopulation and made it display CSCs-like features. It’s interesting and valuable to explore the traits of this subpopulation.

CD133^+^ colorectal cancer cells in both cell line and cancer tissues manifested the characteristics made them special and easier to invade and metastasize. In this study, we found that CD133^+^ HT29 cells showed EMT phenotypes and co-expressed B7H1, indicating CSCs may express B7H1 to evade immune surveillance when they invade through EMT. Similar to HT29 cells, CD133 expression level in colorectal cancer tissues was variable in different cells. Previous studies showed that CD133 was expressed on cancer cells but not haemocytes [4, 5] and most carcinoma cells expressed a membrane glycoprotein EpCAM [34], which is considered as one of CSC markers in several carcinoma types [37].We found that CD133 was expressed on EpCAM^+^ cells (Fig 4), indicating those CD133 expressing cells are colorectal cancer cells. Though EMT is indicated as the characteristic of CSCs, whether there is inherent connection between them remain unclear. In previous studies, CSCs-like cells could be generated by induced EMT activation in cell lines or mouse model [19, 38]. Thus, we thought that similar phenomenon might exist in colorectal cancer. In the current experiment, confocal microscope was used to detect different markers expression in cancers, which could observe several proteins expression in each cell simultaneously. As expected, we found that CD133^+^ cells in human colorectal cancer tissues had EMT phenotypes, downregulating E-cadherin and upregulating vimentin. This phenomenon happened in a portion of CD133 positive cells, which might be concerned with the reason that EMT was a dynamic course and hard to catch the switch. Taken together, our results indicate the direct connection between CSCs and EMT in colorectal cancer tissues.

CSCs have intrinsic biological characteristics to form tumor and may metastasize through EMT. It would be interesting to determine how they evade immune surveillance for final survival especially in immunocompetent hosts. B7H1 is a crucial negative co-stimulatory molecule leading to immunoevasion in cancers. B7H1 expression on CD133^+^ cancer cells indicates that B7H1 may play a role in CSCs. Recently clinical trails showed that blocking PD-1-B7H1 pathway results in relative good prognosis in anti-cancer therapy and B7H1 expression in cancer tissues is associated with outcome of the treatment [39]. Based on what we found here, this remarkable outcome might be related to control CSCs by targeting PD-1-B7H1 pathway. B7H1 is considered a better target than another negative co-stimulating molecule, CTLA-4, because the distribution of B7H1/PD-1 axis is within the tumor microenvironment more selectively [40, 41]. Our results suggest that targeting B7H1 might hurt the “prime culprit” of cancer, cancer stem cells, in colorectal cancer. Moreover, some B7H1^+^ manifested EMT-like phenotype, hinting the potential connection of immunoevasion and EMT. In transgenic mice model, it had been proved that upregulation of B7-H1 in skin epithelial cells promoted EMT and accelerates carcinogenesis [42], which was consistent with our finding that B7H1 expressing cancer cells had EMT phenotypes in colorectal cancer tissues.

Taken together, our results showed B7H1 expression and EMT phenotypes in CD133^+^ cells, indicating the potential mechanisms for CSCs to escape immune surveillance and invade distant tissues. Though some studies doubt about CD133 for CSCs’ marker, our results suggest that in CD133^+^ subpopulation included a section of cells possesses characteristics helping cells invade and metastasize, especially in which manifest EMT phenotypes and express B7H1. Such a small section of cancer cells have powerful tumorigenicity and could invade and metastasize without immune attack, which may close to the theoretic CSCs in functional aspects. Nevertheless, it is still uncertain whether EMT and B7H1 expression of CSCs are two independent events or regulated by the same signaling pathway at the same time. It was shown that mTOR signal and hypoxia-inducible factor -1α, which was an important factor to induce EMT, regulated CD133 expression in cancer cells [43], indicating that CD133 expression and EMT phenotypes were related to hypoxia and might be regulated by mTOR signal. Moreover, B7H1 was regulated by PTEN through the PI3K/AKT/mTOR signaling in pancreatic cancer [44]. These studies suggested that mTOR signaling might be involved in the relationship between EMT and immune evasion in CSCs. Thereafter, we are trying to find out the important pathway concerned with EMT and immunevasion in CSCs in further study, which may be a crucial target in cancer therapy.

## Supporting Information

S1 Fig CD133 is scarcely expressed in paraneplastic tissue. Immunofluorescence of frozen section of colon cancer sample shows that there is a region of relative normal beside cancer nests, where CD133 (red) is rather weaker (left) than in cancer nest (right). DAPI (grey) was used to stain nuclear. It is shown a representative of colon moderately differentiated adenocarcinoma, T3N0M0. (Bar = 50 μm)
(TIF)
