# Supplementary figures and images for: B7H1 Expression and Epithelial-To-Mesenchymal Transition Phenotypes on Colorectal Cancer Stem-Like Cells
Source: PLoS One. 2015 Aug 18;10(8):e0135528. doi: 10.1371/journal.pone.0135528 (PMC4540313; doi:10.1371/journal.pone.0135528)

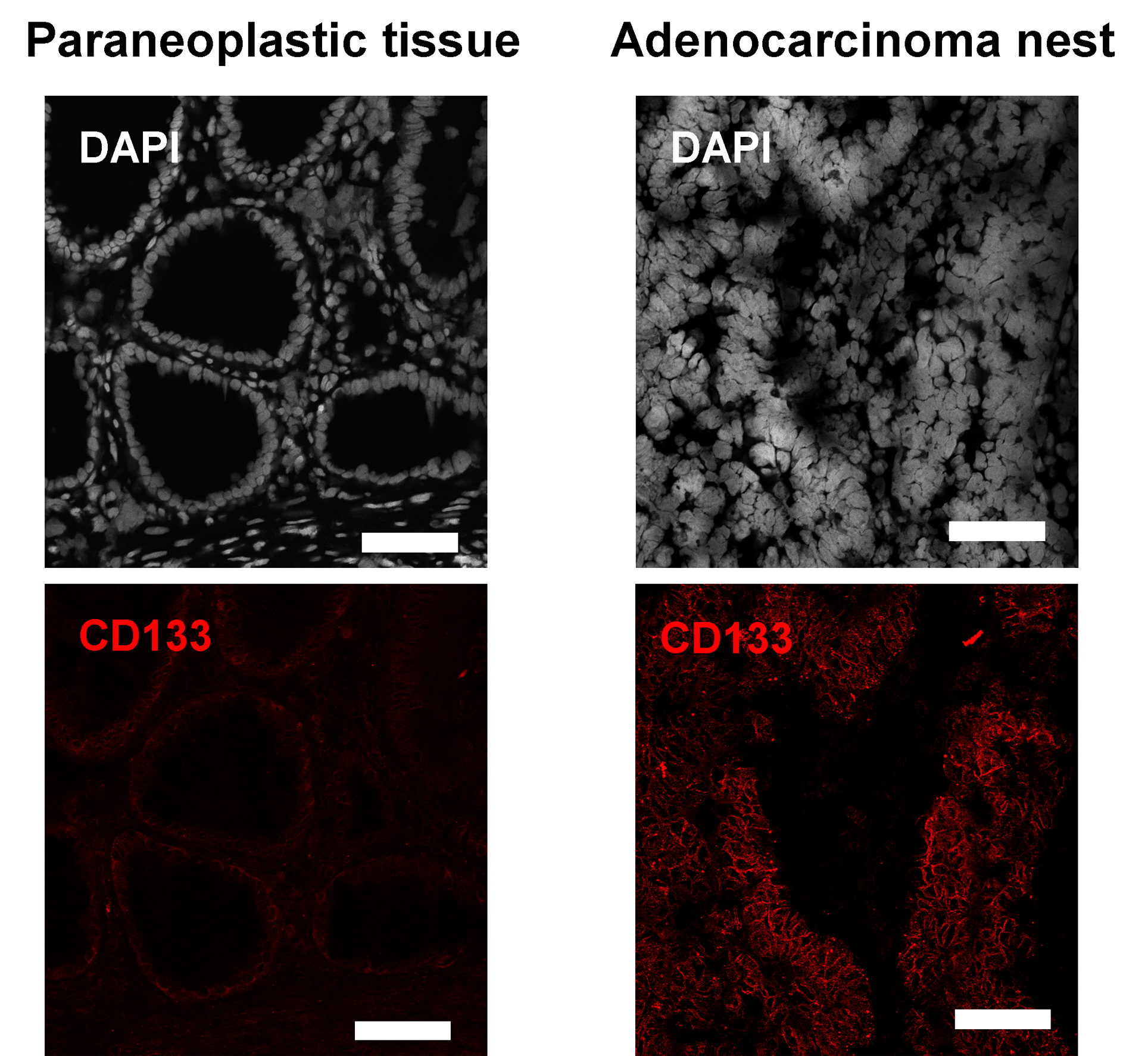

Supplement: S1 Fig — Immunofluorescence of frozen section of colon cancer sample shows that there is a region of relative normal beside cancer nests, where CD133 (red) is rather weaker (left) than in cancer nest (right). DAPI (grey) was used to stain nuclear. It is shown a representative of colon moderately differentiated adenocarcinoma, T3N0M0. (Bar = 50 μm) (TIF) [file pone.0135528.s001.tif]
